# Associations between High-Density Lipoprotein Functionality and Major Adverse Cardiovascular Events in Patients Who Have Undergone Coronary Computed Tomography Angiography

**DOI:** 10.3390/jcm10112431

**Published:** 2021-05-30

**Authors:** Hiroko Inoue, Yuhei Shiga, Kenji Norimatsu, Kohei Tashiro, Makito Futami, Yasunori Suematsu, Makoto Sugihara, Hiroaki Nishikawa, Yousuke Katsuda, Shin-ichiro Miura

**Affiliations:** 1 Department of Cardiology, Fukuoka University Nishijin Hospital, Fukuoka 814-8522, Japan; hiroco61@fukuoka-u.ac.jp (H.I.); kenjimm020010@yahoo.co.jp (K.N.); makito8718@yahoo.co.jp (M.F.); hiroaki@fukuoka-u.ac.jp (H.N.); katsuda@fukuoka-u.ac.jp (Y.K.); 2 Department of Cardiology, Fukuoka University School of Medicine, Fukuoka 814-0180, Japan; kohei.t1027@gmail.com (K.T.); ysuematsu@fukuoka-u.ac.jp (Y.S.); msma93@adm.fukuoka-u.ac.jp (M.S.)

**Keywords:** high-density lipoprotein, cholesterol efflux capacity, major adverse cardiovascular events

## Abstract

The present study aimed to investigate the associations between high-density lipoprotein (HDL) functionality and major adverse cardiovascular events (MACE) in patients who have undergone coronary computed tomography angiography (CCTA). We performed a prospective cohort study and enrolled 151 patients who underwent CCTA and had a follow-up of up to 5 years. We measured cholesterol efflux capacity (CEC), caspase-3/7 activity and monocyte chemoattractant protein-1 (MCP-1) secretion as bioassays of HDL functionality. The patients were divided into MACE(−) (*n* = 138) and MACE(+) (*n* = 13) groups. While there was no significant difference in %CEC, caspase-3/7 activity or MCP-1 secretion between the MACE(−) and MACE(+) groups, total CEC and HDL cholesterol (HDL-C) in the MACE(+) group were significantly lower than those in the MACE(−) group. Total CEC was correlated with HDL-C. A receiver-operating characteristic curve analysis showed that there was no significant difference between the areas under the curves for total CEC and HDL-C. In conclusion, total CEC in addition to HDL-C, but not %CEC, was associated with the presence of MACE. On the other hand, HDL functionality with regard to anti-inflammatory and anti-apoptosis effects was not associated with MACE.

## 1. Introduction

In patients who are treated for atherosclerotic cardiovascular disease (ASCVD), there is a possibility of some residual risks even when the low-density lipoprotein cholesterol (LDL-C) level has been significantly reduced [1,2]. Such residual risks include high levels of the triglyceride (TG), a low level of high-density lipoprotein cholesterol (HDL-C) and other uncontrolled risk factors [1,2,3,4,5,6]. A recent study in a Japanese cohort indicated that extremely high HDL-C (≥90 mg/dL) had an adverse effect on ASCVD mortality [7]. HDL mainly enhances reverse cholesterol transport, such as the cholesterol efflux capacity (CEC), as well as having anti-oxidative, anti-inflammatory and anti-apoptosis functions [8,9,10]. Recently, it has been considered that both HDL quality and HDL quantity are important for preventing CVD. Prospective studies revealed that CEC was inversely correlated with the incidence of CV events [11]. We also reported that the restenosis rates after coronary stent implantation were associated with CEC [12]. Thus, HDL functionality is a critical residual risk factor for ASCVD.

Coronary computed tomography angiography (CCTA) has become more widely available in many general hospitals and enables the accurate non-invasive assessment of coronary artery stenosis for screening of coronary artery disease (CAD). In our previous cross-sectional study, high levels of HDL-C at the time of CCTA were associated with a reduced presence and severity of CAD [13]. In addition, total CEC and HDL-C were associated with the presence of CAD, while %CEC was not [14]. However, that study did not analyze the associations between various HDL functionalities, including CEC and the prognosis. Therefore, we determined the associations in this study.

## 2. Materials and Methods

In our previous study, 204 consecutive subjects who underwent CCTA for screening of CAD and were either clinically suspected to have CAD or had at least one cardiovascular risk factor were enrolled, and CEC was measured [14]. In this study, we excluded 53 of those patients due to the absence of follow-up (*n* = 8) and an insufficient volume of blood samples for further analysis of HDL functionality (*n* = 45), and finally analyzed HDL functionality in 151 patients.

We followed the patients for up to 5 years (average, 3.7 ± 0.7 years) and divided them into those with ((+) group, *n* = 13) and without ((−) group, *n* = 138) a major adverse cardiovascular event (MACE), where MACE was defined as all-cause deaths, acute myocardial infarction, coronary revascularization and ischemic stroke as a composite primary endpoint. When the patients had significant coronary stenosis as assessed by CCTA and received coronary intervention immediately after CCTA, the intervention was not included in MACE as coronary revascularization. We measured HDL functionality including CEC, caspase 3/7 activity associated with apoptosis and the secretion of monocyte chemotactic protein-1 (MCP-1) associated with inflammation. The protocol in this study was approved by the ethics committee of the Fukuoka University Hospital (# 09-10-02). All subjects gave their written informed consent to participate.

### 2.1. Evaluation of Coronary Stenosis Using CCTA

We assessed coronary stenosis using CCTA [13,14]. All patients were scanned by 64-multidetector row CT on an Aquilion 64 (TOSHIBA, Tokyo, Japan). The region of interest was placed within the ascending aorta. The scan was started when the CT density reached 100 Hounsfield Units higher than the baseline density. The scan was performed between the tracheal bifurcation and the diaphragm. All segments were evaluated according to the 15-segment American Heart Association coronary artery model. Fifteen segments of coronary arteries were evaluated. CAD was defined as any narrowing of the normal contrast-enhanced lumen to more than 50% in at least one major coronary artery that could be identified in multi-planar reconstructions or cross-sectional images. The number of significantly stenosed coronary vessels (0, 1, 2 and 3VD) was determined. In addition, the atherosclerotic severity of coronary artery disease was assessed in terms of the Gensini score.

### 2.2. Evaluation of CAD Risk Factors and Left Ventricular Ejection Fraction (LVEF)

Age, gender, body mass index (BMI), systolic blood pressure (SBP), diastolic BP (DBP), smoking status (current vs. nonsmoker), family history (myocardial infarction (MI), angina pectoris or sudden death) and chronic kidney disease (CKD) were collected as risk factors for CAD. Data of serum levels of LDL-C, HDL-C, triglycerides (TG), hemoglobin A1c (HbA1c) and fasting blood glucose (FBG) were also collected. LVEF was assessed by transthoracic echocardiography. BMI was calculated as weight (kg)/height (m^2^). BP was determined as the mean of two measurements obtained in an office setting by the conventional cuff method using a mercury sphygmomanometer after at least 5 min of rest. The presence of dyslipidemia (DL), hypertension (HTN), diabetes mellitus (DM) and use of medication were obtained from medical records. Patients who had SBP ≥ 140 mmHg and/or DBP ≥ 90 mmHg at present or who were taking antihypertensive treatment were considered to have HTN. Patients with LDL-C ≥ 140 mg/dL, TG ≥ 150 mg/dL and/or HDL-C < 40 mg/dL or who were taking lipid-lowering treatment were considered to have DL. Patients with FBG ≥ 126 mg/dL, HbA1c ≥6.5% or who were receiving a glucose-lowering drug were considered to have DM. We calculated the estimated glomerular filtration rate (eGFR) from the data of serum creatinine, age, body size and gender. We defined CKD as when eGFR was less than 60 mL per minute per 1.73 m^2^ body surface area.

### 2.3. Measurement of HDL CEC

We examined HDL CEC with an ex vivo system that used J774 macrophages and HDL isolated from plasma of the study patients by ultracentrifugation [14]. Briefly, J774 macrophages were cultured and radiolabeled with 2 μCi/mL of ^3^H-cholesterol for 24 h. The day after labeling, the cells were washed and incubated with 8-Br-cAMP to upregulate ATP-binding cassette A1 transporter. Efflux medium containing isolated HDL (15 μg) was added for 4 h. Radiolabeled cholesterol counts were analyzed for the cell compartment and media. Percentage (%) of CEC was calculated as follows: (radioactivity in the medium/total radioactivity (radioactivity in medium + cells extracted with NaOH/NaCl)) × 100-CEC in sample-free medium. Total CEC was also calculated as the percentage of cholesterol efflux capacity/100 × HDL-C level.

### 2.4. Measurement of Secretion of Monocyte Chemotactic Protein 1 (MCP-1)

We evaluated the HDL-induced secretion of MCP-1 with an ex vivo system using human coronary endothelial cells (HCECs, Clonetics, San Diego, CA, USA) [15] and apo-B-depleted plasma from the study participants as samples. HCECs were cultured and grown in media. In the experiments, HCECs were washed with medium. The cells were incubated with 5 μg/mL of samples under the same conditions for 24 h. After 24 h, the secretion of MCP-1 in the medium from HCECs was measured by a Human CCL2/MCP-1 Quantikine ELISA Kit (R & D Systems, Minneapolis, MN, USA). The relative secretion of MCP-1 in each sample was calculated by the ratio of the secretion in each sample to the secretion in standard HDL (EMD Millipore Corp., Billerica, MA, USA). The relative total secretion of MCP-1 was also calculated as the relative secretion of the MCP-1/100 × HDL-C level.

### 2.5. Measurement of Caspase 3/7 Activity

We analyzed the HDL-suppressed caspase 3/7 activity with an ex vivo system that used the H9C2 cell line of embryonic rat cardiomyoblasts (ATCC^®^, CRL-1446^T^, Manassas, VA, USA) and apo-B-depleted plasma from the study participants as samples. We used cardiomyoblasts to analyze anti-apoptosis by HDL because HDL may prevent the progression of cardiac dysfunction related to apoptosis. H9C2 cells were cultured and grown in media. In the experiments, H9C2 cells were grown under serum-free conditions for 2 h. After 2 h, the cells were incubated with 5 μg/mL of samples for an additional 6 h. The caspase 3/7 activities in the H9C2 cells were measured by the Caspase-Glo^®^ 3/7Assay System (Promega Corp., Madison, WI, USA). Relative caspase 3/7 activity in each sample was calculated by the ratio of the activity in each sample to the activity in standard HDL. Relative total caspase 3/7 activity was also calculated as the relative caspase 3/7 activity/100 × HDL-C level.

### 2.6. Statistical Analysis

The statistical analysis was performed using IBM SPSS statistics software, version 22 (SPSS Inc., Chicago, IL, USA) and EZR, which is used in R (The R Foundation for Statistical Computing, Vienna, Austria). More precisely, it is a modified version of R Commander designed to add statistical functions frequently used in biostatistics [16]. Continuous variables are shown as mean ± standard deviation. Continuous and categorical variables were compared between the groups by the *t* test and a Chi-square analysis, respectively. We performed a Wilcoxon rank-sum test when continuous variables did not show a normal distribution expressed as a median value and interquartile range. The Spearman rank correlation coefficient was used to evaluate associations between the groups. A receiver-operating characteristic (ROC) curve analysis was used to determine the cut-off of the total CEC or HDL-C to distinguish between with and without MACE at the highest possible sensitivity and specificity levels. Area under the curve (AUC) values were compared between total CEC and HDL-C by a Chi-square analysis. A value of *p* < 0.05 was considered significant.

## 3. Results

### 3.1. Patient Characteristics in All Patients and in the MACE(+) and MACE(−) Groups

Table 1 shows the patient characteristics in all patients and in the MACE(+) and MACE(−) groups. The mean age was 65 (58–71) years and BMI was 23 ± 3 kg/m^2^ in all patients. The frequencies of HTN, DL and DM in all patients were 78%, 71% and 23%, respectively. The MACE(+) group showed a higher level of %smoking and a lower level of HDL-C than the MACE(−) group. There were no significant differences in other factors, including %CAD, the number of VD, Gensini score, left ventricular ejection fraction (LVEF), %CKD, eGFR and medications between the MACE(+) and MACE(−) groups.

### 3.2. %CEC, Total CEC and HDL-C in the MACE(+) and MACE(−) Groups

**Figure 1:**
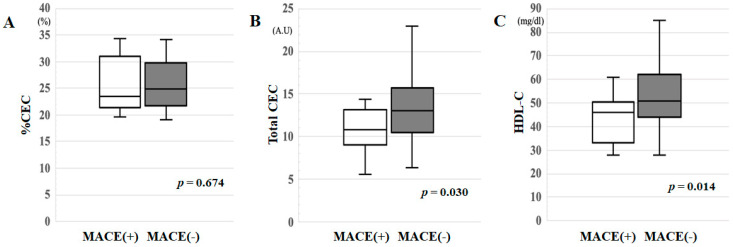
(**A**) %CEC, (**B**) total CEC and (**C**) HDL-C in the MACE(+) and MACE(−) groups. *A.U*: arbitrary unit.

As shown in Figure 1A–C, the MACE(+) group showed significantly lower total CEC (*p* = 0.030) and HDL-C levels (*p* = 0.014) than the MACE(−) group, while there was no difference in %CEC between the groups.

### 3.3. Relative Caspase 3/7 Activity, Relative Total Caspase 3/7 Activity, Relative Secretion of MCP-1 and Relative Total Secretion of MCP-1 in the MACE(+) and MACE(−) Groups

**Figure 2:**
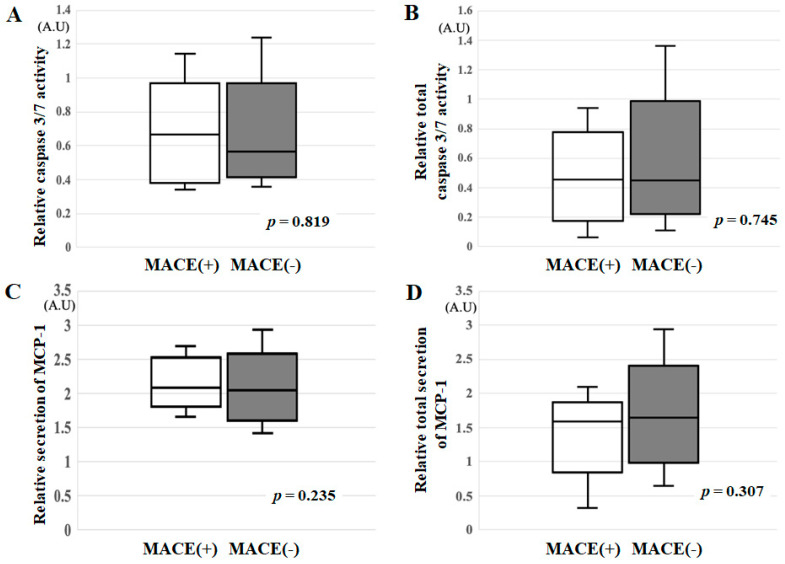
(**A**) Relative caspase 3/7 activity, (**B**) relative total caspase 3/7 activity, (**C**) relative secretion of MCP-1 and (**D**) relative total secretion of MCP-1 in the MACE(+) and MACE(−) groups. *A.U*: arbitrary unit.

Figure 2 shows caspase 3/7 activity and the secretion of MCP-1. There were no differences in relative caspase 3/7 activity (*p* = 0.819), relative total caspase 3/7 activity (*p* = 0.745), the relative secretion of MCP-1 (*p* = 0.235) or relative total secretion of MCP-1 (*p* = 0.307) between the groups.

### 3.4. Correlations between %CEC, Total CEC and HDL-C in All Patients

**Figure 3:**
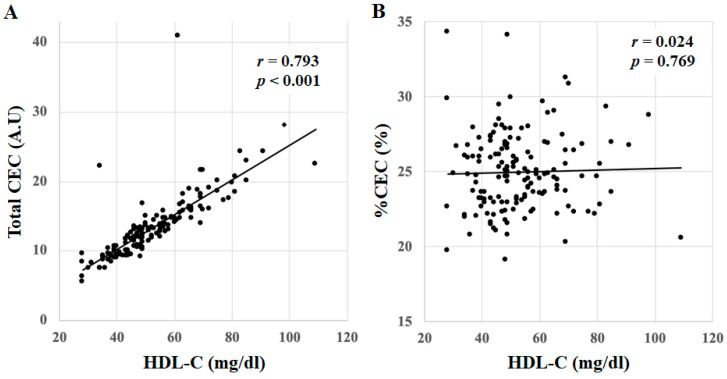
Correlations between (**A**) %CEC, (**B**) total CEC and HDL-C in all patients. *A.U*: arbitrary unit.

Total CEC was positively correlated with HDL-C in all patients (*r* = 0.793, *p* < 0.001), whereas %CEC showed no correlation (*r* = 0.024, *p* = 0.769) (Figure 3A,B).

### 3.5. Cut-Off Values of Total CEC or HDL-C Levels for the Diagnosis of MACE in All Patients

**Figure 4:**
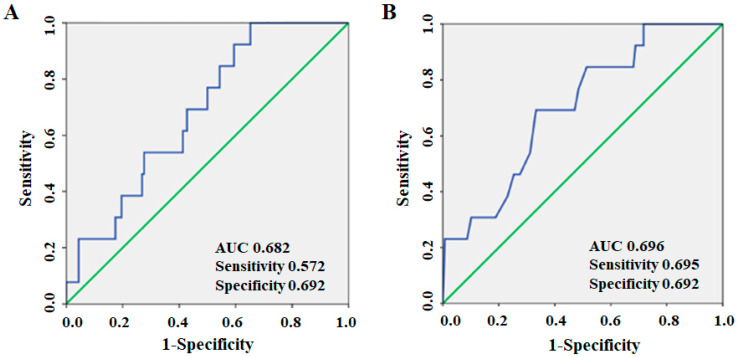
Cut-off values of (**A**) total CEC or (**B**) HDL-C levels for the diagnosis of MACE in all patients.

A ROC curve analysis showed that the AUC for total CEC and HDL-C were 0.682 and 0.696, respectively, in all patients (Figure 4A,B). The cut-off levels of total CEC and HDL-C that gave the greatest sensitivity and specificity for the presence of CAD were 12.4 (sensitivity 0.572, specificity 0.692) and 47 mg/dL (sensitivity 0.659, specificity 0.692), respectively. There was no significant difference between the AUC for total CEC and HDL-C (*p* = 0.656), which indicated that these two factors contributed to MACE to a similar extent.

## 4. Discussion

In this study, we hypothesized that HDL functionality, CEC in particular, was associated with MACE. The main finding was that total CEC and HDL-C in the MACE(+) group were significantly lower than those in the MACE(−) group. In addition, total CEC was correlated with HDL-C. These two factors contributed to MACE to a similar extent. On the other hand, HDL functionality with regard to anti-inflammatory and anti-apoptosis effects was not associated with MACE.

We showed that total CEC and HDL-C in the MACE(+) group were significantly lower than those in the MACE(−) group. We measured %CEC using isolated HDL by ultracentrifugation to eliminate the effects of other lipoproteins as much as possible, since several studies have reported that other lipoproteins might influence cholesterol efflux capacity [17,18]. By this method, we could estimate the efflux capacity for a fixed amount of isolated HDL. This reflects the effect of the per unit capacity of HDL, but not total CEC in the bloodstream. Therefore, %CEC was normalized to total CEC in blood by the HDL-C concentration. Although we used HDL without the effects of other lipoproteins to measure pure CEC, %CEC values alone could not predict the occurrence of MACE. The involvement of other lipoproteins, other than Apo-AI, was also considered. Since the MACE (+) group had significantly lower HDL levels and the total CEC, which is the cholesterol uptake rate multiplied by the HDL-C value, was associated with MACE, the HDL-C value itself was at least related to MACE. In addition, since the correlation coefficient between HDL-C and total CEC was 0.793, which is a relatively strong correlation, it may be possible to predict MACE from the HDL-C value without measuring total CEC. However, the HDL-C value is 20–30% of the weight of HDL, and it is not clear whether the HDL-C value alone directly reflects the functionality of HDL itself. In any case, these results show that both HDL quality and quantity are important. In this study, the cut-off levels of total CEC and HDL-C for the presence of MACE according to a ROC curve analysis were 12.4 and 47 mg/dL, respectively. To the best of our knowledge, only our previous report has addressed the cut-off levels of the total CEC for the diagnosis of CAD [14]. In that study, the total CEC in the presence of CAD was 12.2, which is similar to the value observed here. Next, the cut-off level of HDL-C for the presence of MACE was 47 mg/dL, which is neither high nor low compared to values in the literature [6,19]. The cut-off level of HDL-C for the diagnosis of CAD was 48 mg/dL [14], which is similar to the cut-off for the presence of MACE. In addition, after adjusting for demographics, co-morbidities, lipid profile, statin use and date of procedure, our model demonstrated a U-shaped association between HDL-C and overall mortality, with HDL-C levels of 30–50 mg/dL associated with the most favorable outcomes, and HDL-C levels <30 mg/dL or >50 mg/dL associated with worse outcomes [20]. Decreased HDL-C levels were associated with a significantly increased risk of CV events in women (<49 mg/dL in women) but not in men (<42 mg/dL in men) [21]. According to the Japan Atherosclerosis Society Guidelines for Prevention of Atherosclerotic Cardiovascular Diseases 2017, serum HDL-C levels should be maintained ≥40 mg/dL for the primary and secondary prevention of CVD [6]. Thus, the cut-off level of HDL-C at 47 mg/dL, while relatively low, seems to be reasonable. On the other hand, higher levels of HDL-C have not been found to be associated with atheroprotection [7,22,23,24]. In NIPPON DATA90, high HDL-C (60–79 mg/dL) was associated with a significantly reduced risk of CAD, whereas very high HDL-C (≥80 mg/dL) was not [24]. Recently, extremely high levels of HDL-C (≥90 mg/dL) were significantly associated with an increased risk of ASCVD mortality, an increased risk of CAD and ischemic stroke in a pooled analysis of Japanese cohorts (EPOCH-JAPAN) [7]. In any case, our results suggest that when patients show a total CEC less than around 12.4 and/or HDL-C less than around 47 mg/dL at the time of CCTA, they might develop MACE in the future.

HDL functionality with regard to anti-inflammation and anti-apoptosis was not associated with MACE in this study. Many studies have shown that vascular inflammation is associated with adverse events and C-reactive protein is a critical biomarker of CVD [25,26,27,28,29,30]. HDL mainly acts as a scavenger, removing deposited cholesterol from macrophages. It also provides anti-inflammation and anti-apoptosis effects. Inflammation and apoptosis are associated with not only HDL function, but also with many other inflammation factors (MCP-1 [30], interleukin-6 and -8 [31], etc.) and apoptosis factors (tumor necrosis factor-a, B-cell lymphoma 2 [32], etc.). Thus, anti-inflammation and anti-apoptosis by HDL were not associated with MACE.

This study has several important limitations. First, although the sample size was relatively small, which limited our ability to determine significance, such as in the ROC analysis, including the cut-off levels of total CEC and HDL-C, which may be affected by gender-specific differences, we found that total CEC clearly had a significant correlation with MACE. Second, the population was only selected from Japan and the findings may not be applicable to other populations. Third, the CEC assay itself has several limitations because cell-based assays are labor-intensive. We did not analyze the anti-oxidative function of HDL. Fourth, we divided patients according to the presence of MACE and the duration of follow-up was only up to five years. A large-scale survey with a longer follow-up and further analysis will be needed.

## 5. Conclusions

Total CEC was correlated with HDL-C. Total CEC in addition to HDL-C, but not %CEC, was associated with the presence of MACE. These two factors contributed to MACE to a similar extent. On the other hand, HDL functionality with regard to anti-inflammatory and anti-apoptosis effects was not associated with MACE.

## Figures and Tables

**Table 1:** Patient characteristics in all patients, the MACE(+) group and the MACE(−) group.

	All Patients (*n* = 151)	MACE(+) Group (*n* = 13)	MACE(−) Group (*n* = 138)
Age (years)	65 (58–71)	67 (58–70)	64 (58–71)
Male (%)	62	62	57
BMI (kg/m^2^)	23 ± 3	23 ± 4	23 ± 3
Smoking (%)	39	62 *	34
HTN (%)	78	77	71
SBP (mmHg)	134 (123–146)	136 (123–146)	133 (123–146)
DBP (mmHg)	76 ± 12	79 ± 19	76 ± 11
DL (%)	71	77	64
LDL-C (mg/dL)	108 ± 28	94 ± 28	109 ± 28
HDL-C (mg/dL)	50 (43–61)	46 (33–50) *	51 (44–62)
TG (mg/dL)	114 (79–159)	113 (76–187)	114 (79–156)
DM (%)	23	23	21
HbA1c (%)	5.6 (5.3–6.2)	5.8 (5.4–6.6)	5.6 (5.3–6.2)
FBG (mg/dL)	101 (93–114)	101 (93–124)	101 (93–114)
CKD (%)	5	8	6
eGFR (mL/min/1.73 m^2^)	66 ± 14	67 ± 10	68 ± 14
LVEF (%)	68 ± 9	67 ± 12	68 ± 5
CAD (%)	48	61	47
The number of VD	0.92 ± 1.10	1.38 ± 1.32	0.88 ± 1.07
Gensini score	12 ± 15	16 ± 15	11 ± 14
Medications			
ARB/ACE-I (%)	44	46	41
CCB (%)	35	31	33
β-blocker (%)	17	0	17
Diuretic (%)	13	15	12
Statin (%)	34	46	35
EPA (%)	2	8	2
Insulin (%)	10	8	6
Sulfonylurea (%)	13	23	12
Biguanide (%)	10	23	9
DPP4-I (%)	10	15	9

*MACE:* major adverse cardiovascular events, *BMI:* body mass index, *HTN:* hypertension, *SBP:* systolic blood pressure, *DBP:* diastolic BP, *DL:* dyslipidemia, *LDL-C:* low-density lipoprotein cholesterol, *HDL-C:* high-density lipoprotein cholesterol, *TG:* triglyceride, *DM:* diabetes mellitus, *HbA1c* hemoglobin A1c, *FBG:* fasting blood glucose, *CKD: chronic kidney disease, eGFR:* estimated glomerular filtration rate, *LVEF:* left ventricular ejection fraction, *CAD:* coronary artery disease, *the number of VD:* the number of significant stenosed coronary vessels, *ARB/ACE-I:* angiotensin II receptor blocker/angiotensin converting enzyme inhibitor, *CCB:* calcium channel blocker, *EPA:* eicosapentaenoic acid, *DPP4-I:* dipeptidyl peptidase-4-inhibitor. * *p* < 0.05 vs. MACE(−) group.

## Data Availability

The data that support the findings of this study are available from the corresponding author upon reasonable request.
